# Effectiveness of Breaded Chicken Coated with Whey Protein Isolate on Oil Absorption during Frying in Antioxidant-Rich Frying Oil

**DOI:** 10.3390/foods13060937

**Published:** 2024-03-19

**Authors:** Qi Jin, Abigail Garrett, Robert Brannan

**Affiliations:** 1 Department of Exercise and Nutrition Sciences, Weber State University, Ogden, UT 84403, USA; qijin1@weber.edu; 2 Division of Treatment Services, Safe Haven Ministries, Inc., Clarksville, AR 72830, USA; abigailjanegarrett@gmail.com; 3 Division of Food and Nutrition Sciences, Ohio University, Athens, OH 45701, USA

**Keywords:** oil stabilization, lipid reduction, total polar materials, whey protein isolate

## Abstract

Breaded chicken coated in whey protein isolate (WPI) has been shown to reduce oil absorption during batch frying. What is not known is how this is affected by repeated fryings and whether antioxidant-rich oil will enhance this effect. The objective of this research was to determine how successive daily frying of WPI-coated breaded chicken in antioxidant-rich oil affects oil quality and oil inhibition, moisture retention, color, and texture of the breaded chicken. Chicken fritters with and without a 10% WPI post-breading dip were fried successively for 6 h per day over five days in oil without antioxidant or with either 1000 ppm rosemary extract or propyl gallate. The control oil became spent at 12 h of frying (>24% TPM, <50% DEGLEV). During this time, the oils treated with antioxidants were significantly less spent, and the WPI-treated fritters fried in these oils exhibited 22–49% less lipid; retained 10–18% more moisture; and became darker, less red, and less yellow (lower L*, a*, and b* values) compared to the un-dipped fritters fried in the control oil. These results suggest that the presence of antioxidants in the frying oil mitigated some of the degradative changes in the oil during frying but likely did not play a major role in moisture retention or inhibition of oil absorption.

## 1. Introduction

Deep fat frying is a conventional cooking technique that involves immersing a food in hot oil. Frying increases the caloric content of the food due to oil absorption. The oil absorption mechanism is complicated and cannot be thoroughly explained by any single theory; however, three mechanisms of oil absorption—water replacement, cooling-phase effect, and surfactant theory—can account for most or all of the oil absorbed by fried foods [1]. The water replacement theory describes the substitution of water vapor for hot cooking oil during frying [2] caused by oil migration through pores initiated by surface moisture loss. It has been suggested that the high temperature of frying quickly brings water to its boiling point such that water vapor escapes, leaving voids in the crust region through which oil migrates via uneven pressure distribution from the core and crust [3]. However, fat and moisture transfer is also observed after frying, which is described by the cooling-phase effect. The cooling of the crust after frying condenses remaining water in the substrate and is responsible for a pressure drop that initiates oil absorption [4]. The surfactant theory ties oil uptake to surfactants generated in the frying oil during frying as oil degrades. This mixture of diglycerides, monoglycerides, fatty acids, and glycerol are more polar than fresh oil and act as surfactants that decrease surface tension between the water and the oil, leading to more contact with the oil and the food [1].

The relationship between hydrolytic and oxidative rancidity of frying oil during frying and mechanisms of protection using antioxidants has been extensively reviewed [5,6,7,8]. There is agreement in these reviews that frying oils contain endogenous antioxidants that are either minimized during processing or overwhelmed during the intensity of frying [7]. Thus, researchers have explored the addition of exogenous, phenolic-rich antioxidants to frying oils from a variety of synthetic or plant-based sources. These reviews indicate that the effect of natural, i.e., plant-based, antioxidants is generally weaker than their synthetic counterparts, especially in meat systems, and their use can be limited due to solubility and dispersal issues. Further, many of these studies were performed in model systems that do not include any product being fried, necessitating the need for research that accounts for the complex interactions between the frying oil and the substrates being fried [6].

These complex interactions are more difficult to describe in breaded and fried meat products compared to products that are more compositionally homogenous such as French fried potatoes or tortilla chips. The addition of ingredients such as proteins and hydrocolloids to various steps in the frying process of breaded and fried meat products, described in recent reviews [9,10], has resulted in inhibition of oil absorption to varying degrees. Research from our laboratory has shown that whey protein isolate significantly reduced oil absorption compared to an untreated control when used as a post-breading dip in ground chicken patties [11] and bone-in chicken thighs [10]. Others have shown similar results in chicken strips and chunks [12]. Similar findings were shown using egg albumin solutions as a post-breading dip in fried chicken patties [13,14] and fish balls [5].

To develop an effective strategy to enhance reduction of oil uptake in fried foods, the goal should be to produce a product with an acceptable macronutrient distribution range (AMDR) of fat, in the range of 20–35%, which in most fried foods only could be achieved with a reduction of oil content in the range of 60% [9]. Because our previous research suggests that applying whey protein isolate solution at low pH in the post-breading stage effectively reduces fat uptake in fried chicken fritters to some degree, modification of frying oil by adding an antioxidant could be another method of reducing oil absorption according to the surfactant theory and could work synergistically with our protein-based approach. Thus, our research objective was to determine how successive daily frying of WPI-coated breaded chicken in antioxidant-rich oil affects the oil quality and oil inhibition, moisture retention, color, and texture of the breaded chicken. This study could lead to fried foods that contain less oil and a prolonged frying life of the oil in which they were fried.

## 2. Materials and Methods

### 2.1. Materials

All food ingredients were obtained from local food sources. WPI was donated from Davisco Foods International (Eden Prairie, MN, USA). Food-grade sodium bisulfate (pHase) was donated by Jones Hamilton Co., Walbridge, OH, USA. Rosemary extract, propyl gallate, hexane, and other chemicals were purchased from Fisher Scientific (Fair Lawn, NJ, USA).

### 2.2. Preparation of Chicken “Fritters”

Shown in Table 1, all of the coated patties in this study exceeded 30% total coating pickup by weight, so they are considered “fritters” according to the U.S. Department of Agriculture [15] and will be referred to as such hereafter. For each replication, chicken breasts were ground once through an 8 mm plate using a food processor (model K45SS/250W, KitchenAid^®^, Whirpool Corporation, Benton Harbor, MI, USA), after which uniform 20 ± 2 g patties were made using a 5 cm diameter mold. Patties were frozen (−20 °C) for up to 24 h before coating was applied to aid in processing.

In groups of four, chicken patties were pre-dusted, battered, breaded, and treated with post-breading dip (if required). The pre-dust consisted of all-purpose wheat flour. The batter was prepared as needed and consisted of 48.75% (*w*/*w*) all-purpose flour, 48.75% corn flour, 1.0% xanthan gum, 1.0% salt, 0.5% baking powder, and deionized water [16]. The viscosity of the batter was adjusted using a modified Stein Cup method and was considered adequate when a full 4 in diameter funnel emptied in 11 ± 1 s. The breading consisted of cracker meal that had passed through a 2 mm sieve (Fisherbrand Test Sieve, Fair Lawn, NJ, USA) to eliminate particles larger than the pore size. The post-breading dip consisted of 10% WPI in distilled water and was adjusted to pH 2 using sodium bisulfite. The solution was prepared up to 24 h in advance, stored at 4 °C, and equilibrated to room temperature before use. The chicken patties were weighed before and after each processing step.

Four identical fryers (Presto^®^ Dual ProFryTM/1800W, National Presto Industries Inc., Eau Claire, WI, USA) containing corn oil were equilibrated at 375 °F for at least 30 min before use. Two of the fryers contained corn oil with no antioxidant. One fryer was used only to fry the samples that were not subjected to the WPI dip treatment and were considered the control group (CON). The other three fryers were used for the WPI-dipped patties, one without antioxidant added to the oil (WPI) and the other two with either 1000 ppm rosemary extract (RM) or 1000 ppm propyl gallate (PG) dissolved in the oil.

Four chicken fritters from each treatment were fried at the same time each day and six hours later in the day for five consecutive days. Stress was induced in the oil at 30 min intervals throughout the day by frying four fritters prepared as described above on the hour and 30 g of commercial chicken popcorn (Any’tizers^®^) on the half-hour. Oil samples were taken before the first fritters were introduced to the oil at the beginning of each day and after the last fritters were removed from the oil six hours later and were stored at −40 °C until analyzed. At the end of each day, the fryers were turned off and the oil was left covered overnight. At the beginning of the next day, 200 mL of fresh corn oil was added.

### 2.3. Color and Texture Analysis

Three chicken fritters from each treatment were randomly selected from each fryer after frying and both sides were analyzed for color and texture. The CIE L*, a*, and b* values were collected using a colorimeter (Konica BC-10, Konica Minolta Sensing Americas Inc., Ramsey, NJ, USA) within 5 min after the chicken fritters were removed from the fryer. After the non-destructive color measurement, texture was analyzed on the same fritters using an instrumental texture analyzer Ta-XT2i (Texture Technologies Corp., Scarsdale, NY, USA/Stable Micro Systems, Godalming, Surrey, UK). Each measurement was conducted using a 70 mm knife-blade at a crosshead speed of 10 mm/s to a depth of 5 mm. Both sides of the three separate chicken fritters were analyzed. Exponent v 5.0 software was used to operate the device. Crust fracture work, crust fracture force, total work, and hardness were determined from the force-determination curves obtained from the software output according to a previous method [17].

### 2.4. Lipid and Moisture Analysis

Within 10 min after being removed from the oil, chicken fritters were finely ground using a food processor (Osterizer, Jarden Corp., Boca Raton, FL, USA) at the highest setting for 30 s. Moisture analysis was performed by oven drying until constant weight was obtained. Lipid from dried samples was extracted in hexane using a Soxhlet extraction system.

### 2.5. Oil Quality Analysis

Total polar material (TPM), anisidine value (AnV), and acid value (AV) were determined using FTIR spectroscopy according to a standard method [18]. Oil degradation level (DEGLEV), a statistically derived value that has been developed to monitor the level of oil degradation and to determine the point of disposal of used frying oils, was calculated as DEGLEV = 117 − (8 × AV) − (3 × TPM) [18]. The viscosity (cP) of the oil samples was measured using Brookfield viscometer with #1 spindle at a speed of 20 at 20 ± 1 °C. Conjugated diene (CD) values (mmol/L) of the oil samples were determined following AOCS procedure 1a-64 [19]. An oil sample (1 g) was dissolved in hexane, and absorption of the conjugated dienes was measured at 234 nm and quantified using the molar extinction coefficient of 29,000 mol/L^−1^ cm^−1^. The peroxide value (PV) of the oil samples was determined by titration with sodium thiosulfate according to AOCS Cd 8-53 [20]. PV was calculated as mEq peroxide/kg sample = (S − B) × N × 1000/sample mass, where S is the volume of titrant of the sample, B is the volume of the titrant of the blank, and N is the normality of sodium thiosulfate solution.

### 2.6. Statistical Analysis

All statistical analysis was conducted using SPSS (version 29.0). Processing parameters were analyzed using analysis of variance (ANOVA) and reported as mean ± standard deviation (SD). Duncan’s post hoc test was used separate means between the groups.

The freshly prepared chicken fritters that were assigned to each of the four fryers were fried repeatedly in oil for the five consecutive days of the study. Lipid, moisture, color, texture, and yield of the chicken fritters during frying was analyzed statistically using split-plot repeated measures ANOVA. The six sampling points during the 30 h of continuous frying (0, 6, 12, 18, 24, and 30 h) served as the within-subjects factors, and means for these factors are called omnibus means, also known as “collapsed” means because they are single means generated from each of the four between subject treatment values. The four treatments described previously (CON, WPI, PG, RM) were the between-subjects factors and are reported at each within-subjects factor sampling point, i.e., frying hour.

Measurements of oil degradation (TPM, DEGLEV, viscosity, PV, AnV, CD) during 30 h of frying were analyzed statistically using split-plot repeated measures ANOVA. The same six sampling points over the 30 h sampling period described above served as within-subjects factors, as were the same four between-subjects factors (CON, WPI, PG, RM) described above.

Because the majority of the interactions met the condition of normality, sphericity, and homogeneity for each dependent variable, split-plot repeated measures ANOVA was considered an appropriate statistical tool based on recent research that reports that Type I error and power of the F-statistic are not altered significantly in data with some non-normal interactions [21], and the Huynh–Feldt correction is an appropriate correction when sphericity is not met [21].

## 3. Results and Discussion

The analysis of the effects of WPI as a pre-fry treatment to reduce oil absorption during frying of foods, especially chicken, is well-documented [10,11]. This study reports the relationship between WPI pre-fry treatments and the quality of the frying oil (with and without antioxidants) used during successive daily frying and how the oil absorption and quality parameters of chicken fritters was affected.

### 3.1. Degradation of Oil during Successive Daily Frying

**Figure 1:**
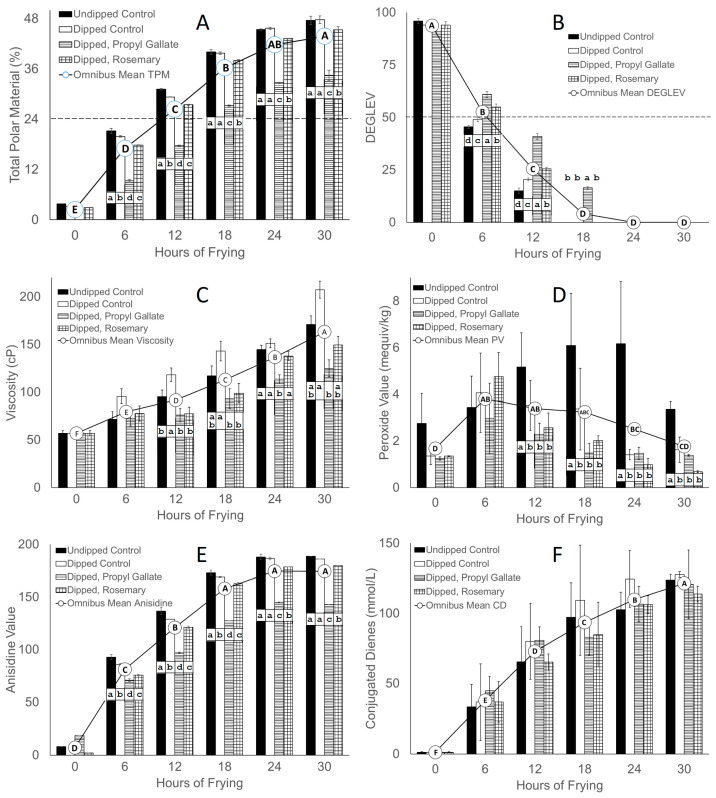
Oil quality measurements ((**A**) total polar material (TMP); (**B**) degradation level (DEGLEV); (**C**) viscosity; (**D**) peroxide value; (**E**) anisidine value; (**F**) conjugated dienes) of frying oil without antioxidant (control) or with rosemary extract or propyl gallate used to repeatedly fry battered and breaded chicken fritters coated without (undipped) or with (dipped) a 10% whey protein isolate post-breading dip over 30 h of frying. Different lowercase letters within a frying hour indicate significant differences (*p* < 0.05) between treatments. Different uppercase letters indicate a significant difference (*p* < 0.05) between the omnibus means across the six sampling periods. Dashed lines indicate 24% TPM and 50% DEGLEV.

The measure of total polar materials (TPM) has long been a standard tool to assess frying oil quality. Germany has set a critical value of 24% TPM in frying oil, with higher values indicating that the oil is spent and should be discarded [22], although the U.S. does not have this type of regulation. Recently, the use of a calculated degradation value (DEGLEV), a statistical manipulation of the acid value and total polar materials, has been offered as an alternative to TPM, with spent oils having a DEGLEV value below 50% [18]. TPM and DEGLEV values are shown in Figure 1A and Figure 1B, respectively. Although the values for TPM and DEGLEV are reported at 6 h intervals, the frying oil was active at 30 min intervals during the 6 h period each day over five consecutive days, so the stress on the oil comes from the continuous frying. The omnibus DEGLEV and TPM means significantly decreased and increased, respectively, for the first 18 h of frying, and all of the oils achieved a DEGLEV value below 50% and three of the four oils achieved TPM greater than 24% by 12 h of frying. At 6 and 12 h of frying, both of the oils treated with antioxidant were significantly “less spent” than their counterparts that were not treated with antioxidant. Erickson et al. [7] reviewed the many studies that have shown a protective effect of rosemary extracts on oil during frying; however, almost all of these studies used homogenous, moisture-controlled frying substrates such as potatoes or dough. Our results agree with results using systems similar to the chicken fritters used in this study. The frying time for TPM to become spent (≈25% TPM) increased from 6 h to 10 h in successive fryings of breaded shrimp in oil with 1000 ppm rosemary oil extract [23] and from 5 h to 9 h in successive fryings of unbreaded chicken wings in oil with 800 ppm rosemary oil extract [24]. There is limited research on the effect of propyl gallate on frying oil other than noting that it is among the least volatile at normal frying temperatures, probably due to the fact that it is a synthetic phenolic compound and its use is regulated in different countries. In any event, we herein report promising results adding to the body of knowledge that adding rosemary extract or propyl gallate to the oil may prolong the life of the oil.

The omnibus mean for frying oil viscosity significantly increased over the 30 h of frying. The data indicate that viscosity of the frying oil was affected by the presence of WPI. Shown in Figure 1C, viscosity was significantly higher at 12 h of frying in the oil without antioxidant used to fry WPI-coated fritters compared to the oil with antioxidant and the oil used to fry fritters without WPI. Taken together with the oil degradation data, these results indicate that in the absence of added antioxidant, the WPI added to the fritters as a post-breading dip likely was responsible for catalyzing the formation of TPM, the concurrent decline in DEGLEV, and the increase in oil viscosity in first hours of frying. The addition of both rosemary extract and propyl gallate to the frying oil significantly mitigated these effects during the same period in spite of the fact that WPI was added to the fritter coating. Nonetheless, these results indicate that the formation of polymers in deep-fat frying, which have long been implicated in the increase in oil viscosity [25], was likely promulgated by WPI and inhibited by the addition of antioxidants to the oil.

Peroxide value (PV), anisidine value (p-AV), and conjugated dienes (CD) also are indicators of oil quality and are shown Figure 1D–F. In regards to the development of PV in the oil, the omnibus mean indicated that there was an initial increase in PV at 6 h of frying with little further change over the frying period. However, all of the frying oil used with WPI-containing fritters exhibited lower PV than the non-WPI control, but the presence of antioxidants in the oil did not significantly affect this result. In similar systems, the PV was reduced by 43% after 10 h of successive fryings of breaded shrimp fried in oil with 1000 ppm rosemary extract [23] and by 40% after 9 h in successive fryings of unbreaded chicken wings in oil with 800 ppm rosemary oil extract [24] compared to their counterparts fried in oil without added antioxidant. Peroxides are not very stable and are known to break down sometime after their formation [26]. In this study, peroxide breakdown could have been exacerbated by the WPI-coated samples, which may not have occurred in the absence of the WPI-coated samples. In regards to the development of AnV in the oil, the omnibus mean indicated that there was a significant increase during the first 18 h of frying, during which time the oil containing antioxidant was significantly lower at each sampling time. This indicates that the presence of the antioxidants in the oil mitigated the formation of AnV. In regards to the development of CD in the oil, the omnibus mean indicated that there was a significant increase throughout frying; however, no differences were observed between the treatments at any of the sampling times. There are no comparable studies comparing the effect of using antioxidant rich frying oils to fry breaded meat products on the formation of AnV or CD.

Of the measurements reported herein, TPM probably is the crucial indicator of fried food quality due to its direct impact on flavor. DEGLEV supports the TPM but is a broader indicator and may not directly translate to specific fried food quality issues. The AnV, PV, and CD are moderately important indicators of oil quality decline. The AnV is an indicator of the presence of aldehydes and ketones that can contribute to off-flavors and aromas in fried food. PV and CD are indicators of the early stages of oil oxidation because peroxides and CD are unstable and are susceptible to further break down into other products that directly affect oil quality. Viscosity is an important measure for maintaining product quality during frying.

### 3.2. Processing Parameters of Chicken Fritters

Processing variables for the unfried chicken were pooled for analysis (by treatment group) regardless of when they were fried so that any differences in raw material before frying for any of the treatments could be identified. Shown in Table 1, all of the coated patties in this study exceeded 30% coating pickup by weight, so they are considered “fritters” according to the U.S. Department of Agriculture [15]. No significant difference was found in raw chicken weight (g), pre-dust pickup (%), batter pickup (%), and breading pickup (%) between the patties assigned to the four treatments. The 10% WPI dip pickup was not significantly different between the treatments to which the dip was applied. The total pickup was affected by the presence of the WPI post-breading dip, and consequently the total pickup of the undipped control was significantly lower than all the other groups.

### 3.3. Oil Absorption and Moisture Retention in Chicken Fritters

**Figure 2:**
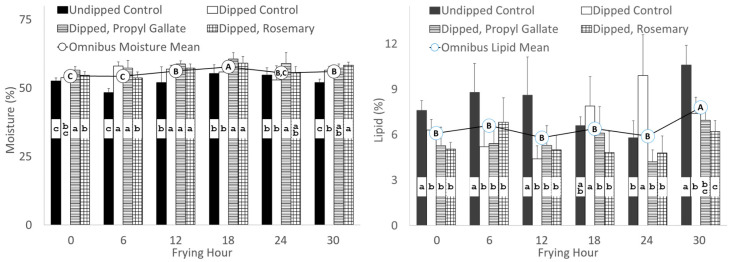
Moisture (**left**) and lipid (**right**) percent of battered and breaded chicken fritters coated without (undipped) or with (dipped) a 10% whey protein isolate post-breading dip in frying oil without antioxidant (control) or with rosemary extract or propyl gallate over 30 h of frying. Different lowercase letters within a frying hour indicate significant differences (*p* < 0.05) between treatments. Different uppercase letters indicate a significant difference (*p* < 0.05) between the omnibus means across the six sampling periods.

Although there were significant differences across the frying period for both moisture and oil content of the chicken fritters (Figure 2), repeated measures ANOVA indicated that total polar materials exceeded 24% and DEGLEV fell below 50% after 12 h of frying, indicating that the oil was spent, so this discussion focuses on that period. For the first 12 h of frying, the omnibus means for moisture increased after 6 h of frying. During that period, repeated measures ANOVA revealed that the moisture content of the fritters coated with the WPI dip and fried in the oil with antioxidant contained 10–18% more moisture (*p* < 0.05) than the control fritters that did not contain the WPI dip and were fried in oil without the addition of antioxidant. We have previously hypothesized that this result probably is due to the thermogelation of the WPI post-breading dip during frying, which is speculated to prevent loss of moisture from chicken fritters [9,13].

Considering that the WPI coating may be preventing the loss of water from the chicken fritters, it is a reasonable hypothesis that the WPI-dipped groups would exhibit lower lipid content. The omnibus means for lipid across the first 18 h of frying did not indicate a significant difference in lipid for the chicken fritters; however, all of the WPI-treated fritters exhibited 22–49% less lipid (*p* < 0.05) compared to the undipped fritters fried in oil to which no antioxidant was added. There were no significant differences in lipid content within a frying hour for the WPI-treated fritters. These moisture and lipid results for WPI-treated fritters are in general agreement with previous research [9]; however, they do not suggest that the presence of antioxidants in the oil worked synergistically to further enhance moisture content or decrease oil content. In other words, these results suggest that the presence of antioxidants in the frying oil that were able to mitigate some of the degradative changes in the oil during frying (Figure 1) did not play a major role in moisture retention or inhibition of oil absorption.

### 3.4. Color and Texture in Chicken Fritters

**Figure 3:**
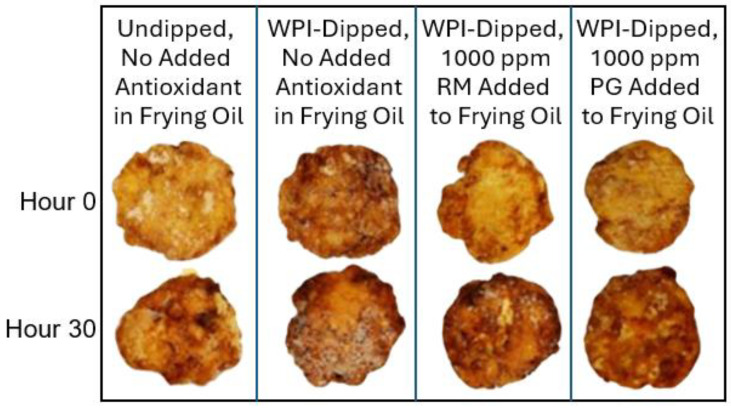
Battered and breaded chicken fritters coated without (undipped) or with (WPI-dipped) a 10% whey protein isolate post-breading dip fried in oil without antioxidant or with rosemary extract (RM) or propyl gallate (PG) that was fresh (Hour 0) and after 30 h of successive frying (Hour 30).

Analysis of the quality of the fritters from a sensory analysis perspective was beyond the scope of this research. However, objective color and texture measurements can provide insight into quality changes during frying and can provide several advantages over relying solely on sensory analysis. Untrained sensory panels are susceptible to biases and can vary based on individual preferences and lighting conditions, whereas specific color and texture parameters are reproducible and can be used to enforce quality control in manufacturing environments and provide benchmarks for compositional and processing factors like frying time and temperature or breading type. Visualization of a sample the chicken fritters produced in this work are shown in Figure 3.

There was a significant difference in the L*, a*, and b* color measurements on the fritters for the omnibus means over the frying period, which showed that during the first 12 h of frying, the fritters became darker, less red, and less yellow (i.e., lower L*, a*, and b* values). However, as shown in Table 2, the initial frying (0 h) of the fritters dipped in WPI exhibited significantly lower L* and higher a* and b* values compared to the undipped control. In other words, the WPI-treated fritters were more dark, red, and yellow compared to the undipped control fritters after the initial frying. No significant differences were found between any of the WPI-treated fritters. In a previous study conducted in our lab [27], similar results were found for L* and a*. The darker and redder appearance of chicken fritters can be explained by non-enzymatic Maillard reaction or caramelization due to the presence of reducing sugars produced from hydrolysis of carbohydrate from the breading and protein from the WPI [27,28].

The darker and redder appearance of chicken fritters can be explained by non-enzymatic Maillard reaction or caramelization due to the presence of reducing sugars produced from hydrolysis of carbohydrate from the breading and protein from the WPI [27,28].

Four instrumental texture measurements (hardness, crust fracture force, crust work, and total work) were monitored. There was a significant difference in the hardness and total work on the fritters for the omnibus means over the frying period, which showed that during the first 12 h of frying, the fritters became harder, and the total work increased. However, as shown in Table 3, there was only a significant difference between the treatments for hardness and total work after the initial frying (0 h), and these results were somewhat ambiguous. This suggests that the changes to the oil during frying rather than the compositional changes in the fritters, i.e., the increase in moisture migration out of the substrate and oil absorption into the substrate, probably explain why texture was affected during frying.

Overall, the WPI treatment but not the presence of antioxidants in the frying oil appeared to be the driving force for the increase in darkness, redness, and yellowness in the fritters, and the degradation of the oil appeared to be the driving force affecting the texture of the fritters. What is not known is whether these changes would be apparent to consumers.

### 3.5. Implications on Product Quality, Marketability, and Sustainability

This study affirms that reducing the oil content in breaded chicken can be achieved using a post-breading WPI dip, with implications for product quality, marketability, and sustainability. Reducing oil absorption in fried foods could lead to a rethinking of the flavor of fried foods if lowering the oil content reveals more of the natural flavors of the food, especially delicate flavors that can be masked by the flavor of the frying oil. As consumers become more health-conscious, reduced oil fried foods may be viewed as healthier alternatives rather than an occasional indulgence. These products also could expand into new markets such as health care or school settings. With respect to sustainability, less oil absorbed by the food is potentially less oil wasted, translating to better product utilization for producers. This could also impact the amount of waste oil that would need to be disposed.

### 3.6. Conclusions

A central question of this research was whether the addition of antioxidants to the frying oil would work synergistically with the WPI, which has been shown to inhibit oil absorption during frying. Successive frying of WPI-containing fritters in oil with the addition of either rosemary extract or propyl gallate up to the point the oil was spent (>24% TPM, <50% DEGLEV) did not improve moisture retention or oil inhibition compared to the WPI-dipped control group that was not fried in oil that contained antioxidants. However, there was some indication that the presence of antioxidants in the spent frying oil at 18 and 24 h did enhance moisture retention and oil inhibition in WPI-dipped fritters compared to those fried in the absence of antioxidants (Figure 2). This may indicate a future research direction for rosemary extract or propyl gallate at levels higher than what was employed in this study.

This research holds significance for two key reasons. Firstly, it paves the way for developing lower-fat fried breaded chicken products, aligning with consumer preferences for healthier options. Secondly, it contributes valuable knowledge about oil degradation in oils used to fry complex foods like breaded chicken. Most existing research focuses on simpler, uniform products like potatoes or tortillas. This study highlights a gap in our understanding of how oils break down when frying breaded meats, and future research can address this by focusing on such non-homogeneous food systems.

## Figures and Tables

**Table 1:** Processing variables of unfried battered and breaded chicken fritters treated with (dipped) or without a 10% whey protein isolate post-breading, randomly assigned into one of four frying categories: to be fried in oil with or without (control) rosemary extract or propyl gallate.

Processing Variables	Undipped Control	Dipped Control	Dipped Rosemary	Dipped Propyl Gallate
Raw chicken weight (g)	19.5 ± 1.2	19.6 ± 1.5	19.6 ± 1.4	19.5 ± 1.4
Pre-dust pickup (%)	4.2 ± 1.3	4.6 ± 1.5	4.5 ± 1.1	4.8 ± 0.9
Batter pickup (%)	33.8 ± 4.0	32.6 ± 5.3	33.8 ± 4.6	32.8 ± 3.9
Breading pickup	4.8 ± 1.2	5.2 ± 0.9	4.9 ± 0.9	5.2 ± 1.0
Dip pickup (%)	0.0 ^b^ ± 0.0	2.9 ^a^ ± 1.1	2.8 ^a^ ± 1.3	2.8 ^a^ ± 1.4
Total pickup (%)	45.9 ^b^ ± 4.9	50.2 ^a^ ± 5.5	50.8 ^a^ ± 5.2	50.5 ^a^ ± 5.6

Note. Different letters within the same row indicate a significant difference at *p* < 0.05.

**Table 2:** CIE color values (L*, a*, b*) of battered and breaded chicken fritters treated with (dipped) or without a 10% whey protein isolate post-breading dip in frying oil with or without (control) rosemary extract or propyl gallate over five days of frying.

Hours of Frying	Undipped Control	Dipped Control	Dipped Rosemary	Dipped Propyl Gallate
Lightness (L*)				
0	53.1 ± 4.4 ^a^	46.8 ± 7.7 ^b^	42.9 ± 7.7 ^c^	46.5 ± 4.8 ^b^
6	53.8 ± 4.9	51.4 ± 6.0	52.5 ± 5.0	52.3 ± 4.9
12	54.7 ± 5.0 ^a^	51.2 ± 5.5 ^b^	52.8 ± 5.1 ^a,b^	51.8 ± 4.7 ^b^
18	57.6 ± 3.7 ^a^	52.1 ± 4.8 ^b^	51.8 ± 4.3 ^b^	53.4 ± 5.1 ^b^
24	58.7 ± 3.7 ^a^	54.2 ± 4.0 ^b^	54.2 ± 3.6 ^b^	52.8 ± 4.3 ^b^
30	57.8 ± 5.8 ^a^	51.2 ± 5.8 ^b^	53.1 ± 4.6 ^b^	51.6 ± 2.9 ^b^
Redness (a*)				
0	12.6 ^b^ ± 3.8	17.5 ± 4.6 ^a^	18.3 ± 3.9 ^a^	18.3 ± 3.0 ^a^
6	11.0 ± 3.1 ^b^	12.7 ± 4.8 ^a,b^	13.0 ± 3.3 ^a^	13.1 ± 4.2 ^a^
12	10.6 ± 2.9 ^b^	13.1 ± 4.1 ^a^	13.5 ± 4.7 ^a^	13.1 ± 3.5 ^a^
18	9.5 ± 2.1 ^b^	13.0 ± 3.6 ^a^	13.0 ± 3.5 ^a^	12.7 ± 3.2 ^a^
24	7.4 ± 2.9 ^b^	13.1 ± 3.9 ^a^	11.0 ± 3.1 ^a^	12.2 ± 2.7 ^a^
30	7.5 ± 2.3 ^a^	11.7 ± 2.7 ^a^	11.9 ± 3.1 ^a^	12.4 ± 2.1 ^a^
Yellowness (b*)				
0	30.3 ^b^ ± 14.5	40.9 ^a^ ± 6.6	39.1 ± 6.0 ^a^	43.3 ± 1.9 ^a^
6	27.7 ± 14.7	29.5 ± 15.5	31.5 ± 14.5	31.0 ± 14.6
12	31.4 ± 14.3	29.8 ± 16.1	31.9 ± 14.2	31.5 ± 14.3
18	32.1 ± 11.7	31.5 ± 13.8	31.4 ± 14.3	31.7 ± 13.5
24	30.6 ± 12.0	34.4 ± 10.4	32.9 ± 12.5	33.4 ± 13.2
30	30.1 ± 15.8	29.7 ± 15.6	31.5 ± 15.2	32.7 ± 14.6

Note. Different letters within the same row indicate a significant difference at *p* < 0.05.

**Table 3:** Instrumental texture of battered and breaded chicken fritters treated with (dipped) or without a 10% whey protein isolate post-breading dip in frying oil with or without (control) rosemary extract or propyl gallate over five days of frying.

Hours of Frying	Undipped Control	Dipped Control	Dipped Rosemary	Dipped Propyl Gallate
Hardness (kg)				
0	0.966 ^b^ ± 0.147	1.132 ^a^ ± 0.133	1.059 ^a,b^ ± 0.041	1.060 ^a,b^ ± 0.136
6	0.968 ± 0.182	0.987 ± 0.050	1.042 ± 0.223	1.002 ± 0.245
12	0.902 ± 0.147	0.974 ± 0.108	1.047 ± 0.155	0.959 ± 0.127
18	0.767 ± 0.069	0.814 ± 0.115	0.908 ± 0.205	0.879 ± 0.138
24	0.774 ± 0.147	0.973 ± 0.125	0.864 ± 0.119	0.892 ± 0.111
30	0.792 ± 0.051	0.989 ± 0.093	0.910 ± 0.027	0.841 ± 0.068
Crust fracture force (kg)				
0	0.723 ± 0.093	0.788 ± 0.166	0.658 ± 0.055	0.767 ± 0.068
6	0.743 ± 0.133	0.669 ± 0.127	0.735 ± 0.267	0.681 ± 0.249
12	0.596 ± 0.184	0.727 ± 0.248	0.795 ± 0.188	0.709 ± 0.251
18	0.605 ± 0.091	0.677 ± 0.185	0.572 ± 0.220	0.698 ± 0.213
24	0.538 ± 0.018	0.759 ± 0.226	0.687 ± 0.170	0.535 ± 0.280
30	0.621 ± 0.096	0.759 ± 0.166	0.668 ± 0.122	0.696 ± 0.065
Crust work (kg·s)				
0	0.314 ± 0.081	0.308 ± 0.104	0.236 ± 0.014	0.335 ± 0.047
6	0.297 ± 0.081	0.243 ± 0.092	0.278 ± 0.157	0.258 ± 0.128
12	0.218 ± 0.114	0.300 ± 0.176	0.307 ± 0.122	0.300 ± 0.138
18	0.221 ± 0.064	0.276 ± 0.143	0.176 ± 0.135	0.316 ± 0.145
24	0.184 ± 0.044	0.326 ± 0.179	0.270 ± 0.136	0.207 ± 0.152
30	0.212 ± 0.086	0.306 ± 0.125	0.218 ± 0.121	0.286 ± 0.046
Total work (kg·s)				
0	0.705 ^b^ ± 0.129	0.867 ^a^ ± 0.118	0.811 ^a,b^ ± 0.081	0.812 ^a,b^ ± 0.016
6	0.742 ± 0.155	0.694 ± 0.092	0.698 ± 0.163	0.735 ± 0.242
12	0.653 ± 0.132	0.651 ± 0.147	0.745 ± 0.092	0.673 ± 0.110
18	0.451 ± 0.097	0.494 ± 0.157	0.574 ± 0.343	0.591 ± 0.256
24	0.544 ± 0.148	0.690 ± 0.117	0.560 ± 0.097	0.669 ± 0.117
30	0.473 ± 0.076	0.703 ± 0.100	0.538 ± 0.142	0.549 ± 0.065

Note. Different letters within the same row indicate a significant difference at *p* < 0.05.

## Data Availability

The original contributions presented in the study are included in the article, further inquiries can be directed to the corresponding author.
